# The Possible Antecedents and Consequences of Matching of Food Intake: Examining the Role of Trait Self-Esteem and Interpersonal Closeness

**DOI:** 10.3389/fpsyg.2015.01920

**Published:** 2015-12-22

**Authors:** Elizabeth Hirata, Gerine M. A. Lodder, Ulrich Kühnen, Sonia Lippke, Roel C. J. Hermans

**Affiliations:** ^1^Bremen International Graduate School of Social Sciences, University of Bremen and Jacobs UniversityBremen, Germany; ^2^Department of Sociology, Interuniversity Center for Social Science Theory and Methodology (ICS), University of GroningenGroningen, Netherlands; ^3^Department of Psychology and Methods, Jacobs University BremenBremen, Germany; ^4^Developmental Psychopathology Department, Behavioural Science Institute, Radboud UniversityNijmegen, Netherlands

**Keywords:** eating behavior, matching, self-esteem, interpersonal closeness, food intake

## Abstract

Although there is evidence that people tend to match their intake to that of others, less is known about the motives underlying this effect. The current study, therefore, examined the relationship between self-esteem, a specific factor that has been related to the likelihood of social matching. Further, we examined the effects of food matching on interpersonal closeness among eating companions. The sample included 89 female dyads. All dyads had free access to palatable snack food during a 15 min interaction. For each dyad the matching score was calculated, as well as both individual's trait self-esteem scores and interpersonal closeness with their eating partner. The overall degree of matching within dyads was high, replicating the findings of previous research. No relationship, however, was found between trait self-esteem and the degree of matching. Furthermore, there was no effect of matching on perceived interpersonal closeness with or liking of the other person. These results suggest that self-esteem might not be a robust predictor of matching and that matching of food intake may not result in increased perceived interpersonal closeness or liking among eating partners.

## Introduction

Almost everyone has been through the experience of eating a substantial amount of food and later regretting the decision to have done so. Scientific research has identified many factors that could influence individuals' eating decisions. These factors can range from basic physiology and energy needs to cultural and environmental determinants (e.g., Axelson, 1986; Leibowitz and Alexander, 1998; Rozin, 2005; Rankinen and Bouchard, 2006; Macht, 2008). A particular important role, however, seems to be played by the social context in which the food is consumed (Herman et al., 2003). To date, numerous studies have been conducted on the influence of the presence of others on food intake (see Cruwys et al., 2015, for a review). These studies have consistently shown that social norms about eating have a powerful effect on eating behavior (Higgs, 2015).

The influence of the presence of others on eating has been studied under somewhat different conceptual and methodological approaches. The most dominant line of research within this area is social modeling. These studies most often use an experimental design in which the intake of one co-eater (i.e., the confederate) is predetermined by the experimenter. By doing so, the researchers can directly test whether the participant adjusts her intake upward or downward in line with that of the other person (Cruwys et al., 2015; Vartanian et al., 2015). Likewise, some studies have focused on the intradyadic similarity between eating partners in the amounts consumed; an effect that is known in the literature as social matching (Herman et al., 2005). Finally, researchers have investigated mimicry effects on food intake, a process that refers to the direct copying of the co-eater's bites or sips (Hermans et al., 2012; Bevelander et al., 2013; Sharps et al., 2015). In the context of the present study, we are particularly interested in matching effects on food intake.

Although extensive research has been carried out on how people's food intake is affected by that of others (c.f. Cruwys et al., 2015; Herman, 2015; Vartanian, 2015), less is known about how eating with others might affect social outcomes. One important feature of social eating (i.e., eating with others) is that it could foster bonding or increase social closeness between co-eaters (Fischler, 2011; Neely et al., 2014). For example, research has shown that people who were eating a meal together evaluated their eating partners more positively than those who were not eating together (Aan Het Rot et al., 2015). Within the context of social matching, it has also been proposed that people match their intake to that of the eating companion in order to enhance the social bond with their eating partner (e.g., Hermans et al., 2009a; Robinson et al., 2011; Exline et al., 2012). Thus, people might follow the intake of the other person in order to affiliate or being liked by them. This assumption, however, has not been empirically tested so far. The first aim of the present study, therefore, is to examine the relationship between food matching and interpersonal closeness between eating partners.

A specific personal trait that might moderate matching effects on food intake is self-esteem (Robinson et al., 2011; Cruwys et al., 2015). As Leary et al. (1995) proposed in their sociometer theory, self-esteem can be seen as a monitor of social acceptance, with low self-esteem signaling low perceived social acceptance, which predisposes people to adapt their behavior in order to prevent social devaluation and social exclusion. Consequently, people with lower levels of self-esteem have a higher need to affiliate with others than those with high self-esteem (Leary et al., 1995). Indeed, there is research that suggests a relationship between self-esteem and social matching. Robinson et al. (2011) found that undergraduate students who scored low on self-esteem were more likely to eat the same amount as their eating companion; a finding that was explained by their increased need for social acceptance. A second aim of the current study, therefore, is to attempt to replicate the findings of Robinson et al. (2011) and investigate whether trait self-esteem indeed affects the magnitude of the matching effect among female undergraduate students.

Altogether, the current study was conducted to gain more insight into the relationship between self-esteem and food matching and the link between food matching and interpersonal closeness among co-eaters. Specifically, we examined whether self-esteem, a trait related to the need for affiliation, is related to the degree of matching. In an attempt to replicate the findings of Robinson et al. (2011), we first examined whether dyadic self-esteem increased matching. In addition to the original study, we investigated the direction of the effects; by testing whether a dyad member with a low self-esteem score was more likely to match the food intake of their high self-esteem counterpart, or vice versa. Furthermore, we used the Actor-Partner-Interdependence-Model (APIM) for indistinguishable dyads (Olsen and Kenny, 2006) to test whether matching of food-intake is related to interpersonal closeness. This is a more accurate analytical strategy for dyadic data, as it accounts for non-independence of food intake and interpersonal closeness among dyad members (Cruwys et al., 2015). To test these ideas, we conducted a dyadic study in which participants were offered free access to palatable snack food.

## Methods

### Participants

The total sample consisted of 89 female dyads. All participants were between 17 and 30 years of age (*M* = 20.32; *SD* = 2.21) and had a mean BMI of 22.57 (*SD* = 2.93). The study protocol was approved by the IRB of the Faculty of Social Sciences, Radboud University, Nijmegen and all participants gave their informed signed consent, in accordance with the Declaration of Helsinki, before they took part in the actual experiment. All participants agreed to being videotaped and approved the use of these data for research purposes. Their participation was granted with either credit points or a financial reimbursement. See Table 1 for participants' characteristics.

**Table 1:** **Descriptive statistics for the full sample**.

	***N***	***M***	***SD***
Age (years)	178	20.32	2.21
BMI	176	22.57	2.93
Hunger	178	3.86	1.95
Sweets craving	178	3.67	2.64
M&Ms consumed	178	4.34	7.77
Self-esteem	178	3.24	0.51
Interpersonal closeness	178	3.28	1.47

### Design and procedure

Participants were invited to the laboratory under the pretext of a study about evaluation of movie trailers. This was a cover story to distract participants from the actual aim of the experiment (i.e., matching effects on food intake).

The experimenter met both participants at the main entrance of the laboratory facilities and accompanied them to the room were the experiment took place. The facility was decorated as an ordinary living room in order to create an ecological-valid research setting (c.f. Hermans et al., 2009b). Their first task was to fill in the consent form and a pre-measure of hunger and craving for sweets. Next, participants watched and evaluated three movie trailers, based on a few bogus questions (e.g., “This trailer made me curious about the movie”; “I would like to see the entire movie”). At this point, they were requested not to communicate with each other. This task took approximately 6 min. After this evaluation task, participants were told that they had a short break in which they were asked to discuss the movie trailers and their favorite movies. In order to create an enjoyable atmosphere, the experimenter left a pitcher of water and a bowl full of chocolate-coated peanut M&Ms. The bowl was within arm's reach of both participants. The experimenter told both women that they were free to help themselves to water and M&M's, and left them alone for 15 min. The break was video-recorded by an unobtrusive camera hidden in the corner of the room and the experimenter coded both participants' eating behavior in a room adjacent to this laboratory. After 15 min, the experimenter re-entered the room and invited one of the participants to come with her to another room to complete a set of questions. By doing so, both participants had enough privacy to fill in the questionnaires. This questionnaire included measures of trait self-esteem, interpersonal closeness, evaluation of the break, dietary restraint, and evaluation of interpersonal closeness with and liking of the interaction partner. After participants completed the questionnaire, the experimenter measured their height and weight. Finally, they were debriefed, thanked and dismissed.

### Materials and measures

#### Food intake and matching

The total quantity of food consumed by each participant (i.e., single pieces of M&M's) was used as our dependent variable. Most of the participants picked one M&M at a time making it possible to accurately count the number of M&Ms consumed during the break. If this was not the case, the experimenter re-watched the videos to accurately count the number of M&Ms. Matching of food intake was determined by the absolute difference between both dyad members' M&M's intake. For example, if one partner ate 5 units of M&M's and her counterpart ate 3, their resulting matching score was 2. The lower this score, the higher the dyadic matching (c.f. Robinson et al., 2011).

#### Familiarity with the other person

Participants' familiarity with each other was assessed with the question “Did you know the other person before the beginning of the experiment?” (yes/no).

#### Self-esteem

Participants' trait self-esteem was measured with the Rosenberg's self-esteem scale (Rosenberg, 1965). The scale consists of 10 items answered on a 4 point likert scale (e.g., “On the whole, I am satisfied with myself”; “I feel that I have a number of good qualities”) 1 = strongly disagree, 4 = strongly agree. Cronbach's alpha was 0.88. Dyadic self-esteem was determined by averaging self-esteem scores of both dyad members.

#### Perceived interpersonal closeness with and liking of the interaction partner

Participants' perceived closeness was measured after the break, with the Inclusion of Other in the Self scale (IOS; Aron et al., 1992). This is a single-item, pictorial measure, which depicts two circles varying in 7 degrees to which they overlap with each other. Greater overlap means higher perceived closeness with the other person. In addition, we investigated whether the degree of matching influenced how much the interaction partners liked each other. To do so, we asked participants how much they liked the other participant, after the break, with a VAS varying from 1 (not at all) to 13.5 cm (very much).

#### Weight and height

Participants' weight was measured with a digital scale (Seca Bella 840, Seca GmbH & co. kg., Hamburg, Germany) and their height was measured with a stadiometer attached to the wall (Seca 206, Seca GmbH & co. kg., Hamburg, Germany).

#### Hunger

In order to control for the potential confounding effect of hunger on intake, we measured participants' hunger level before the break with a 10 point scale ranging from 1 (not at all hungry) to 10 (very hungry; Hermans et al., 2008).

#### Craving for sweets

In order to control for the potential confounding effect of craving on food intake, participants' craving for sweets was measured with a single item scale, similar to a VAS, in which participants were required to mark in a continuous line how much they perceived to be craving sweets at that moment. The scale was fully subdivided, ranging from 0 (no craving at all) to 100 (very high craving).

### Analytic strategy

In accordance with Robinson et al. (2011), we first performed a regression analysis to predict matching of food intake from dyadic self-esteem. Although this analysis is suitable to test the direct relation between dyadic self-esteem (i.e., the average self-esteem within the dyad) and matching of food intake at a dyadic level, it does not allow for testing within dyads. That is, it does not give insight into the question whether the person with the lowest self-esteem score is more likely to match her counterpart's eating behavior than the person with the highest self-esteem score. To examine this, we used a regression analysis, by predicting the food intake of the partner with the lower self-esteem score from the food intake of the other person with a higher self-esteem score. To test whether the dyad member with low self-esteem is indeed likely to match the food intake of their high self-esteem counterpart, we included an interaction between the self-esteem score of the low-self-esteem dyad member, and the food intake of the high self-esteem dyad member. This analysis allowed us to examine whether low self-esteem dyad members match the intake of their interaction partners, and whether this matching is stronger in dyad members with lower self-esteem.

The possible relationship between matching and dyad members' perceived interpersonal closeness was analyzed by using an Actor-Partner-Interdependence-Model (APIM) for indistinguishable dyads (Olsen and Kenny, 2006). This model can be used to estimate three effects. First, it allows the estimation of the effect of food intake on dyad members' own evaluation of interpersonal closeness (actor effect). Second, food intake of the interaction partner can be related to participants' evaluation of interpersonal closeness (partner effect). Third, the interaction between participants' eating behavior and their partners' eating behavior can be related to both dyad members' evaluation of interpersonal closeness, to examine the effects of matching on interpersonal closeness (actor by partner interaction effect; cf. Kenny and Cook, 1999). We used Mplus version 6.2 for these analyses, using the maximum likelihood estimator with robust standard errors (MLR). All predictors were centered before analyses. STDYX standardized results are reported here. Because both dyad members evaluated the same relationship regarding interpersonal closeness, dyad members' evaluations are not independent of each other. The APIM model for indistinguishable dyads was designed to take this type of interdependency into account, by constraining the paths of the actor effect, the partner effect, and the actor-by-partner interaction on interpersonal closeness to be equal between dyad members, and by allowing both dyad members' food intake, and interpersonal closeness evaluations to be correlated. The same model was used for investigating if matching related to participant's ratings of how much they liked each other.

## Results

### Descriptives

In total, 89 dyads took part in the current study. In 30 dyads both dyad members ate at least one M&M and in 25 dyads only one member ate any M&M's. Thirty-four dyads consisted of two members who did not eat any chocolate. On average, participants consumed 4.34 M&M's (*SD* = 7.77). Participants in 63 dyads reported that they were unfamiliar with each other, whereas participants in 22 dyads reported that they were familiar with each other before taking part in the study. In four dyads, one participant claimed that she knew the other participant, but this was not reciprocated. For all models, we examined whether results remained similar if we only included dyads in which both interaction partners indicated that they did not know each other before the experiment. The direction and significance of all paths remained the same, regardless on whether or not the dyads with familiar participants were included. Therefore, we report the effects with all dyads combined.

The overall degree of matching within dyads was high, with an intraclass correlation of M&M's intake of 0.55, *p* < 0.001. However, there were dyads in which both dyad members did not consume any M&M's. This resulted in a perfect matching score, because there was no difference in food intake in these dyads. One could argue that matching of non-eating is not the same as matching of eating. Therefore, we repeated analyses including only dyads in which at least one person ate M&M's. The direction and significance of all paths, however, did not change depending on wether these dyads were in- or excluded.

Because hunger (*r* = 0.21, *p* = 0.006) and craving for sweets (*r* = 0.25, *p* = 0.001) significantly correlated with participants' food intake, all analyses were run with and without controlling for these variables. The direction and significance of all paths did not change depending on whether these covariates were in- or excluded. Therefore, we report the more parsimonious model without controlling for these covariates.

Furthermore, a significant correlation was found between participants' individual level intake and their perceived interpersonal closeness with the interaction partner, (*r* = 0.21, *p* = 0.004), which could suggest that the closer participants felt to the other person, the more they ate (and vice versa). However, once tested within the APIM, which accounts for the correlations between dyad members' food intake and interpersonal closeness, this “actor effect” of food intake in IOS was no longer significant. In the coming sections we describe these results in more details. A positive correlation was also found between interpersonal closeness and liking of other person, (*r* = 0.66, *p* < 0.001), indicating that the closer people felt to the other person the more they liked them as well.

### The relationship between trait self-esteem and matching

The first regression analysis revealed no significant relation between dyadic self-esteem and matching, [β = −0.02, *p* = 0.82; adjusted *R*^2^ = −0.01, *F*_(1, 87)_ = 0.05, *p* = 0.82]. Next, we examined whether food intake of the low self-esteem dyad member was predicted by the food intake of their high self-esteem counterpart, by their own low scores on the self-esteem scale, and by the interaction between these two effects. The absolute mean difference in self-esteem between dyad members was of 0.69 (*SD* = 0.47).

A significant interaction effect could indicate that individuals with lower self-esteem are more likely to match the eating behavior of their counterpart, than individuals with higher self-esteem. In four dyads (4.5%), both dyad members had the same self-esteem scores, therefore, these dyads were excluded from the analyses. Results indicated that whereas the high self-esteem dyad member's food intake predicted food intake of the low self-esteem dyad member (β = 0.55, *p* < 0.001), there was no effect of the low self-esteem dyad member's self-esteem score (β = 0.09, *p* = 0.31), or the interaction between self-esteem and food intake (β = −0.04, *p* = 0.80). This indicates that whereas we did find evidence for matching of food intake between dyad members, reflected by the significant main effect of food intake, the matching was not related to self-esteem, reflected by the non-significant interaction effect. Model fit was sufficient for the entire model [adjusted *R*^2^ = 0.31, *F*_(3, 81)_ = 13.80, *p* < 0.001]. Conversely, when examining matching behavior of the high self-esteem dyad member, results indicated that the intake of the high self-esteem dyad member could be predicted from the food intake of the low self-esteem dyad member (β = 0.50, *p* < 0.001), but not from her own self-esteem score (β = −0.04, *p* = 0.67) nor from the interaction between her self-esteem score and the intake of the low self-esteem dyad member (β = 0.11, *p* = 0.32). Thus, high self-esteem dyad partners also matched their intake to that of their (low self-esteem) dyad partners, but this was not related to their self-esteem.

### The relationship between matching and perceived interpersonal closeness with and liking of the interaction partner

**Figure 1:**
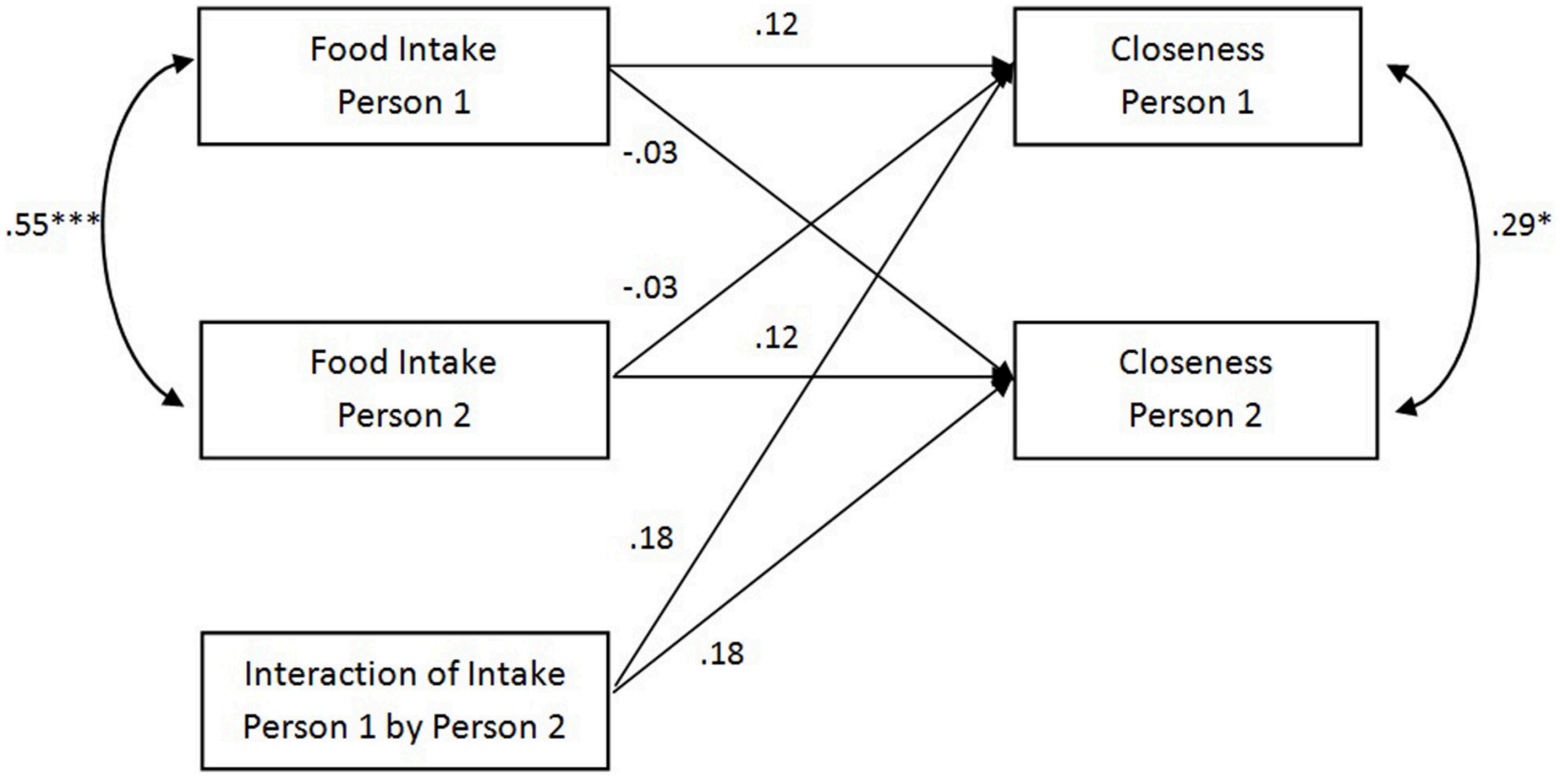
**Results for the APIM model for closeness including actor effects, partner effects, actor-by-partner interactions and intraclass correlations**. ^*^p < 0.05; ^***^p < 0.001.

**Figure 2:**
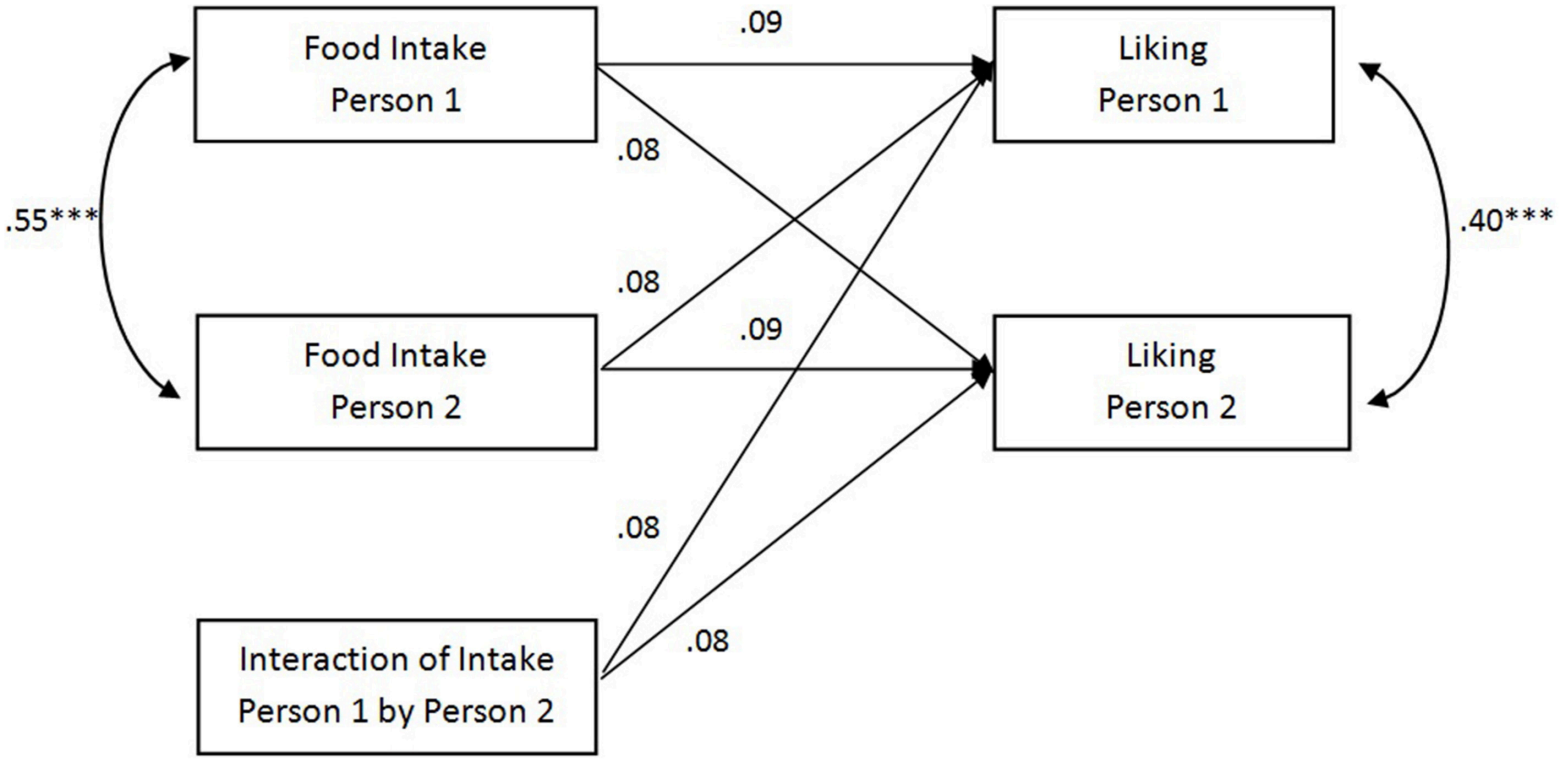
**Results for the APIM model for liking including actor effects, partner effects, actor-by-partner interactions and intraclass correlations**. ^***^p < 0.001.

Next, we used an APIM model for indistinguishable dyads to examine whether matching of food intake predicted interpersonal closeness and liking (Olsen and Kenny, 2006). Results for these analyses are depicted in Figures 1, 2.

The model showed that dyad members were similar in the amount of food they consumed (β = 0.55, *p* < 0.001) and in their evaluations of interpersonal closeness (β = 0.29, *p* = 0.01), with an absolute mean difference between their IOS' scores of 1.32 (*SD* = 1.08). Food intake of participants was neither related to their own evaluation of interpersonal closeness (β = 0.12, *p* = 0.24), nor to their counterparts evaluation of interpersonal closeness (β = −0.03, *p* = 0.80). Furthermore, there was no effect of matching on interpersonal closeness, as evidenced by the non-significant actor-by-partner interaction effect (β = 0.18, *p* = 0.20). This indicates that there is no evidence for the effect of matching of food intake on interpersonal closeness. Due to these non-significant effects, model fit for the entire model was not adequate (RMSEA = 0.11 [90% confidence interval 0.02, 0.19], CFI = 0.57).

In addition, we examined whether matching of food intake was related to liking the interaction partner (RMSEA = 0.09 [90% confidence interval 0.00, 0.17], CFI = 0.68). Results were comparable to those on interpersonal closeness, and showed that dyad members held similar evaluations of each other (β = 0.40, *p* < 0.001), with a small absolute mean difference between their scores, (*M* = 1.50, *SD* = 1.30) and ate similar amounts of food (β = 0.58, *p* < 0.001). Ratings of liking were unrelated to individuals' own food intake (β = 0.09, *p* = 0.41), their interaction partner's food intake (β = 0.08, *p* = 0.34) or matching of food intake, as evidenced by a non-significant actor-by-partner interaction (β = 0.08, *p* = 0.48). Thus, for both interpersonal closeness and liking our results indicated that although participants matched each others' food intake, this matching did not relate to interpersonal closeness or increased liking of the interaction partner.

Finally, as previous research has shown that differences in weight status could affect modeling of food intake (Johnston, 2002; Hermans et al., 2008), we controlled for possible effects of BMI among the dyad members. Our results showed that there were no main actor effects (β = 0.03, *p* = 0.65) or partner effects (β = −0.06, *p* = 0.35), but a significant actor by partner interaction for BMI (β = 0.22, *p* = 0.004), indicating that participants felt closer to each other if they were more similar to each other in terms of BMI. Yet, the size and direction of other effects remained identical when controlling for dyad members' BMI scores, suggesting that differences in BMI were unrelated to the effect of matching on perceived closeness.

## Discussion

Although, there is evidence that people tend to match their intake to that of others, less is known about the motives underlying this effect and its possible social outcomes. To foster this understanding, the current study examined the relationship between self-esteem, a specific factor that has been related to the extent of matching, as well as the effects of food matching on interpersonal closeness and liking of the interaction partner.

Our findings indicated that people tend to eat similar amounts as their co-eater, which is in line with previous studies on matching (e.g., Herman et al., 2005; Salvy et al., 2007; Robinson et al., 2011). No relationship, however, was found between self-esteem and matching of food intake. Furthermore, no evidence was found for a link between matching and interpersonal closeness and liking of the interaction partner. In the following sections, we discuss these results in more detail, in light of the current literature, and present possible explanations for our findings.

### The relationship between self-esteem and matching of food intake

The current study did not replicate the findings of Robinson et al. (2011) who found that self-esteem predicted the degree of matching between eating partners. A possible explanation for the difference in findings between both studies is that there is a statistically significant difference in average self-esteem scores in both samples (tested with a *t*-test using both studies N, M, and SD); participants in our sample had considerably higher self-esteem scores than those in the Robinson et al. study. If it is indeed the case that low self-esteem predicts matching, then participants' relatively high levels of self-esteem, in combination with a relatively low variance in self-esteem scores, could have reduced the possibility to find an effect. Besides, the difference in self-esteem between dyad members appeared to be relatively low. It is possible that, in our sample, these low differences in self-esteem overshadowed any possible effects of this trait on matching. Lastly, Robinson et al. (2011) used a median split to examine matching in dyads containing at least one dyad member with low self-esteem, compared to matching in dyads containing two members with high self-esteem. Although this method is suitable to examine whether matching is more likely to occur if at least one dyad member has low self-esteem scores, it is not suitable to draw conclusions about the direction of the matching effect. That is, this method does not allow a direct test of the hypothesis that participants with low self-esteem match the eating behavior of participants with high self-esteem scores, instead of vice versa. Moreover, the use of median splits leads to a considerable loss of the variance accounted for by the original variable (Cohen, 1983). To solve this issue, we used an analytic procedure that can predict whether the food intake of a low self-esteem dyad member could be predicted from the food intake of a high self-esteem dyad member, and that this prediction would be dependent on the level of self-esteem of the low self-esteem dyad-member. Using this analytic procedure, we did not find support for the assumption that self-esteem predics the degree of matching. On basis of the current study, therefore, we conclude that self-esteem might not be such a robust predictor of food matching. This assumption is supported by the findings of a recent study conducted by Spanos et al. (2015), who demonstrated that individuals' self-esteem did not predict their (self-reported) tendency to eat in response to social cues (Spanos et al., 2015). Further, our findings are in line with previous studies that have failed to identify potential moderators of the matching or modeling effect (e.g., Goldman et al., 1991; Herman et al., 2005; Robinson et al., 2013; Hirata et al., 2015). Given the limited number of studies assessing the relationship between matching and self-esteem, however, further investigation of whether and how self-esteem relates to matching of food intake, or social influences on food intake in general, is strongly recommended.

Future studies aiming at investigating the possible role of trait self-esteem in matching of food intake, for example, may benefit from pre-selecting participants based on their trait self-esteem scores and compose dyads based on these scores (e.g., low vs. high scores and low vs. low scores) and directly compare these dyads on matching behavior. Although, this is a time consuming procedure for researchers, it increases the chance to accurately test the role of self-esteem because the number of individuals with low and high self-esteem scores is predetermined and therefore the variability of self-esteem scores within the study population could be increased.

### The relationship between matching of food intake, interpersonal closeness, and liking

The current study examined whether higher matching of food intake was related to higher levels of interpersonal closeness with and liking of the interaction partner, which were significantly correlated to each other. However, none of these measures related to matching of food intake. A possible explanation for this lack of effect is that other facets of the social interaction taking place during the eating situation might have been responsible for these shifts in connectedness mentioned in previous studies. For example, communication between people occurring during eating episodes might be a strong factor. While eating, people might feel more connected to each other through engaging in a pleasant conversation. Additionally, perceived personal similarities, rather than overlap in eating behavior, might foster the feeling of closeness (Agnew et al., 2004). While beyond the scope of the current study we found, in our own data, that similarities in BMI increased perceived closeness. Furthermore, it is possible that, mimicry, and not matching, might account for differences in interpersonal closeness after the eating episode (e.g., Chartrand and Bargh, 1999; Chartrand and Lakin, 2013). Future research, therefore, could address whether mimicry of food intake leads to increases in unconscious expressions of closeness. Finally, it is also possible that the context in which the food was consumed might not have been strong enough to elicit different feelings of closeness among eating partners. For example, it may be that matching of snack food does not lead to greater interpersonal closeness, whereas matching of food intake during the context of a meal does enhance feelings of connectedness. Given the social function of meal intake in everyday life, it is possible that different effects may be observed when people eat a complete meal together. Future research could, for instance, focus on the question whether different eating contexts (e.g., meals vs. snacks) could induce feelings of closeness and liking among eating partners.

A few limitations should be mentioned. First, we measured individual's interpersonal closeness and liking of their eating partner only after the interaction. We decided not to measure closeness and liking before the interaction, in order to avoid making participants (too) suspicious of the actual aims of the study, as this might negatively impact their natural eating behavior (Robinson et al., 2014). Further, asking them about their closeness with the other person before they had the chance to start interacting might have made them much more aware and critical toward each other, affecting their future interaction. Finally, the design of the current study did not allow us to investigate the possible interaction between self-esteem and initial closeness. Future, research could investigate if initial closeness might interact with self-esteem, in that those lower in self-esteem might be especially motivated to match, when they feel less close to the other participant.

Despite these limitations, this study once again demonstrated that people tend to match their food intake to that of their co-eater. Although, on basis of one single study it cannot be ruled out that self-esteem and interpersonal closeness are important factors within the context of matching effects on eating, we do not find support for the notion that self-esteem is a robust predictor of matching effects on food intake. Furthermore, our findings suggest that matching of food intake might not always reflect an attempt to affiliate with the eating companion, which adds to previous findings of matching occurring even in situations in which it is unlikely that individuals would be strategically seeking to ingratiate themselves with others (e.g., Roth et al., 2001; Burger et al., 2010). Given that research has been increasingly acknowledging the importance of social influences on food intake, further research focusing on the possible antecedents and consequences of matching of food intake may shed light on the question of why people tend to adapt their intake to that of their eating companions.

## Author contributions

Briefly, EH was involved in formulating the research question, designing the study, writing the protocol, collecting and analyzing the data and writing the article. RH was involved in formulating the research question, designing the study, writing the protocol, and writing the article. GL was involved in analyzing the data and writing the article. UK and SL were involved in formulating the research question and writing the article. All authors were responsible for drafting and approving the final manuscript. Lastly, all authors agree to be accountable for all aspects of the work in ensuring that questions related to the accuracy or integrity of any part of the work are appropriately investigated and resolved.

## Funding

This research was funded by a grant from the German Research Foundation (DFG) to the Bremen International Graduate School of Social Sciences (BIGSSS) [grant number: GSC 263/1]. The DFG had no role in the study design, collection, analysis or interpretation of the data, writing the manuscript, or the decision to submit the paper for publication.

### Conflict of interest statement

The authors declare that the research was conducted in the absence of any commercial or financial relationships that could be construed as a potential conflict of interest.
